# HIV-1 Antiretroviral Drug Resistance Mutations in Treatment Naïve and Experienced Panamanian Subjects: Impact on National Use of EFV-Based Schemes

**DOI:** 10.1371/journal.pone.0154317

**Published:** 2016-04-27

**Authors:** Yaxelis Mendoza, Juan Castillo Mewa, Alexander A. Martínez, Yamitzel Zaldívar, Néstor Sosa, Griselda Arteaga, Blas Armién, Christian T. Bautista, Claudia García-Morales, Daniela Tapia-Trejo, Santiago Ávila-Ríos, Gustavo Reyes-Terán, Gonzalo Bello, Juan M. Pascale

**Affiliations:** 1 Direction of Research and Technological Development, Gorgas Memorial Institute for Health Studies, Panama City, Panama; 2 Department of Biotechnology, Acharya Nagarjuna University, Guntur City, India; 3 Department of Genetics and Molecular Biology, School of Biology, University of Panama, Panama City, Panama; 4 Institute for Scientific Research and High Technology Services of Panama, Panama City, Panama; 5 Department of Microbiology, School of Medicine, University of Panama, Panama City, Panama; 6 Facultad de Ciencias de la Salud, Universidad Interamericana de Panamá, Panama City, Panama; 7 Centre for Research in Infectious Diseases, National Institute of Respiratory Diseases (Centro de Investigación en Enfermedades Infecciosas, Instituto Nacional de Enfermedades Respiratorias), Mexico City, Mexico; 8 Laboratório de AIDS e Imunologia Molecular, Instituto Oswaldo Cruz, FIOCRUZ, Rio de Janeiro, Brazil; Institute of Molecular Genetics IMG-CNR, ITALY

## Abstract

The use of antiretroviral therapy in HIV infected subjects prevents AIDS-related illness and delayed occurrence of death. In Panama, rollout of ART started in 1999 and national coverage has reached 62.8% since then. The objective of this study was to determine the level and patterns of acquired drug resistance mutations of clinical relevance (ADR-CRM) and surveillance drug resistance mutations (SDRMs) from 717 HIV-1 *pol* gene sequences obtained from 467 ARV drug-experienced and 250 ARV drug-naïve HIV-1 subtypes B infected subjects during 2007–2013, respectively. The overall prevalence of SDRM and of ADR-CRM during the study period was 9.2% and 87.6%, respectively. The majority of subjects with ADR-CRM had a pattern of mutations that confer resistance to at least two classes of ARV inhibitors. The non-nucleoside reverse transcriptase inhibitor (NNRTI) mutations K103N and P225H were more prevalent in both ARV drug-naïve and ARV drug-experienced subjects. The nucleoside reverse transcriptase inhibitor (NRTI) mutation M184V was more frequent in ARV drug-experienced individuals, while T215YFrev and M41L were more frequent in ARV drug-naïve subjects. Prevalence of mutations associated to protease inhibitors (PI) was lower than 4.1% in both types of subjects. Therefore, there is a high level of resistance (>73%) to Efavirenz/Nevirapine, Lamivudine and Azidothymidine in ARV drug-experienced subjects, and an intermediate to high level of resistance (5–10%) to Efavirenz/Nevirapine in ARV drug-naïve subjects. During the study period, we observed an increasing trend in the prevalence of ADR-CRM in subjects under first-line schemes, but not significant changes in the prevalence of SDRM. These results reinforce the paramount importance of a national surveillance system of ADR-CRM and SDRM for national management policies of subjects living with HIV.

## Introduction

Antiretroviral therapy (ART) has succeeded in achieving long-term suppression of human immunodeficiency virus (HIV) replication, with a consequent reduction of clinical manifestations of infection, as well as prevention or delayed the onset of acquired immune deficiency syndrome (AIDS) [1, 2]. Approximately 15.8 million infected people are currently receiving ART worldwide [3]. In 2012, the number of adults living with HIV infection in Panama was estimated between 18,000 and 20,000 (0.7% prevalence) [4]. In that same year, 9,966 people were eligible for ART, of which only 6,411 (ART coverage of 64%; 260 were children under 15 years old) were receiving antiretroviral (ARV) drugs at no cost [5].

In Panama, ART was rolled out in 1994 with the use of ARV azidothymidine (AZT) as mono-therapy. Between 1994 and 1999, mono-therapy and double nucleoside therapy were used [6]. In 1999, triple therapy (two nucleosides plus a protease inhibitor) was started in the Social Security system [6]. The case-fatality rate of AIDS decreased substantially since 2001 when HIV/AIDS subjects without Social Security had access to ART [4, 7]. In 2007, the Ministry of Health (MINSA) established the first national guidelines on care and treatment for adults following World Health Organization (WHO) recommendations of treating individuals with CD4 cells counts <350 cells/μl [8]. First-line ART for adults included two nucleoside (NRTI) and one non-nucleoside (NNRTI) reverse transcriptase inhibitors [8]. Protease inhibitors (PI) were included in the second-line schemes in 2005; and the integrase inhibitor, Raltegravir, in 2010 [9]. In 2011, MINSA published new guidelines recommending the use of Tenofovir (TDF) with Lamivudine (3TC) or Emtricitabine (FTC) as first-line schemes, and two NRTIs with a Ritonavir-boosted protease inhibitor, usually Lopinavir/Ritonavir (LPV/r), as second-line schemes [10].

The occurrence of ARV drug resistance mutations both in viruses from individuals under ART and in transmitted viruses to ART-naïve individuals is increasing a global level [1, 11]. A study performed during the 2004–2005 evaluating the prevalence of surveillance drug resistance mutations (SDRMs) and acquired drug resistance mutations in ARV drug-naïve and ARV drug-experienced Panamanian subjects, respectively, found that only treated subjects (9.7%, 8/82) harbored mutations conferring high or intermediate resistance levels to ARV drugs [12]. The only study conducted in 2011 among ARV drug-naïve recently-infected subjects, estimated a prevalence of TDRM of 12.8% (6/47) in Panama [13]. These studies, however, were based on the analysis of a limited number of individuals (n < 100) and current prevalence and patterns of ADR-CRM and SDRM in Panama are unknown.

HIV-1 subtype B is the predominant clade in Panama, although other subtypes and circulating recombinant forms (CRF), such as CRF12_BF, CRF20_BG and CRF01A/G, have been described, but remain at low frequency [14, 15]. Phylogenetic studies reveal that HIV-1 subtype B seems to be evolving geographically, mainly by adaptation to different human populations and depending on the mode of HIV transmission [16, 17]. Currently, there are at least three recognizable HIV-1 subtype B lineages identified by their genetic differences, time and region of origin: B_CARIBBEAN_, B_PANDEMIC_ and subtype B´ from Asia [16–19]. We recently identified that the B_PANDEMIC_ and B_CARIBBEAN_ viral lineages coexist in Panamanian HIV infected population [20]. The objective of this study was to determine the prevalence and patterns of drug resistance mutations associated with ART in 717 ARV drug-naïve and drug-experienced HIV-1 subtype B infected subjects from Panama analyzed between 2007 and 2013.

## Materials and Methods

### Study population

The Gorgas Memorial Institute for Health Studies (ICGES) located in Panama City provided 1,179 HIV-1 *pol* sequences from drug-resistance genotyping tests performed in participants referred to the ICGES by infectious disease physicians from June 2007 to December 2013. The epidemiological and clinical information from each subject was extracted retrospectively from the drug-resistance genotyping test form used to request this test. Only infectious disease physicians are licensed by the Ministry of Health to request HIV testing and conduct the clinical management of subjects. The classification of subjects into ARV drug-experienced and ARV drug-naïve was then defined by an infectious disease physician through clinical evaluation and so annotated in the drug-resistance genotyping test form. ARV drug-experienced subjects included all subjects using ARVs regardless of treatment interruptions; whereas, drug-naïve subjects included all subjects without previous exposure to ARV drugs, although the recently-infected states were unknown. Unfortunately, the samples used to perform the drug-resistance genotyping test were not available, and then the “recently-infected” status of ARV drug-naïve subject were not investigated as the World Health Organization (WHO) recommended for surveillance methods in 2008 [21]. To avoid any unrecognized ARV exposure in ARV drug-naïve, each subject was evaluated by checking whether a previous sample was received for any of the diagnostic tests (HIV proviral, CD4+ T cell count or HIV RNA viral load test) required for the clinical management of participants and performed in ICGES since 2001. From the 1,179 sequences that were available, 717 (60.8%) sequences from the first HIV resistance genotype done were selected for this study. The sequences excluded belongs to seven (0.6%) subjects with non-B subtypes that were previously identified [14], nine (0.8%) subjects with missing drug resistance genotyping test form and to 379 (32.1%) subjects with a repeated HIV genotype done over the years. To avoid any bias on drug resistance mutational analysis, 67 (5.7%) subjects infected through mother-to-child transmission were also excluded. This study was approved by the Research Bioethics Committee IRB of the Gorgas Memorial Institute for Health Studies for which verbal informed consent was obtained for all participants safeguarding their rights and privacy. The constructed database was analyzed anonymously and only for epidemiological purposes.

### Genetic characterization of HIV-1 sequences

The complete protease (PR) and the first part of the reverse transcriptase (RT) (nucleotides 2253 to 3275 of reference strain HXB2) were amplified and sequenced as previously described [13, 14]. Subtype assignment of HIV-1 Panamanian sequences was confirmed using phylogenetic and recombination analysis as previously described [14]. To classify subtype B *pol* sequences into subtype B viral lineages, the 717 Panamanian sequences were aligned with those subtype B Panamanian sequences generated during 2004–2005 (n = 132) [12] and with references sequences representative of the B_PANDEMIC_ and the B_CARIBBEAN_ clades described previously [20, 22]. Maximum Likelihood (ML) phylogenetic trees were then inferred under the GTR+I+Γ nucleotide substitution model selected using the jModelTest software [23] and reconstructed as previously described [20].

### ARV drug resistance mutation interpretation analyses

In order to estimate the potential impact of ARV drug resistance-associated mutations on ART response, the Panamanian HIV-1 subtype B *pol* sequences were analyzed using the HIVdb Program Version 7.0 at the Standford HIV Drug Resistance Database (analysis performed on May 22^nd^, 2014) (http://sierra2.stanford.edu/sierra/servlet/JSierra). Acquired drug resistance mutations (ADRM) were evaluated according to the IAS-USA drug resistance mutation list in ARV drug-experienced subjects and only clinically relevant mutations (ADR-CRM), mutations with amino acid substitution conferring resistant, were considered [24]. Drug resistance mutations in ARV drug-naïve subjects were classified and interpreted according to the list of mutations for surveillance drug resistance mutations (SDRMs), version 2009 [25].

### Statistical analyses

Categorical variables were compared using the chi-square test, the Fisher’s exact test, or the Fisher-Freeman-Halton test. For linear trend analysis of binary data the chi-square test was used. Associations were expressed as odds ratios and estimated using the logistic regression model. The statistical programs StatsDirect v2.7.9 (Atrincham, UK) and Stata v 13.1 (Stata Corporation, TX, USA) were used for data analyses. Missing data were excluded when compared percentages. Reported p-values were two-sided and p-values less than 0.05 were considered statistically significant.

### Nucleotide Sequence Accession Numbers

The Panamanian HIV-1 subtype B *pol* sequences have been deposited in GenBank with access numbers KJ473994-KJ474641.

## Results

### Epidemiological and clinical data of HIV-1 subtype B infected subjects

Of the 717 subjects, 467 (65%) were categorized as ARV drug-experienced and 250 (35%) as ARV drug-naïve subjects (Table 1). In both groups, the majority of subjects were males (≥ 67%), residents from eastern Panama (≥ 62%) and considered themselves heterosexual (≥ 66%). The ARV drug-experienced and drug-naïve groups were significantly different for most demographic and clinical variables, except for gender (*p* = 0.907) and plasma viral load concentration (*p* = 0.200). Among ARV drug-experienced subjects, 94% were older than 25 years, 96% had more than 1 year of being diagnosed and 60% displayed CD4+ T cell count less than 200 cells/μl. Regarding ARV drug-naïve subjects, 41% were younger than 24 years, 80% were newly diagnosed (≤ 1 year) and 75% had a CD4+ T cell count greater than 200 cells/μl. We also found that 52% of the ARV drug-experienced subjects had AIDS and that the majority of ARV drug-naïve subjects (92%) were asymptomatic (*p* < 0.001) at time of sampling for drug-resistance genotyping test.

**Table 1:** Epidemiological data of the study cohort in Panamanian ARV drug-experienced and ARV drug-naïve HIV-1 subtype B subjects.

Feature	TOTAL	ARV drug-experienced	ARV drug-naive	
	N = 717 (%)	n = 467 (%)	n = 250 (%)	*p-*value
**Gender**				
Male	480 (67)	311 (67)	169 (68)	0.907
Female	237 (33)	156 (33)	81 (32)	
**Age group (years)**				
≤ 24	130 (18)	27 (6)	103 (41)	**<0.001**
25–44	421 (59)	301 (64)	120 (48)	
45–76	166 (23)	139 (30)	27 (11)	
**Geographic location [n = 712]**				
East of Panama Province	474 (67)	288 (62)	186 (75)	**0.008**
West of Panama Province	113 (16)	86 (19)	27 (11)	
Colon Province	58 (8)	43 (9)	15 (6)	
Other provinces	67 (9)	46 (10)	21 (8)	
**HIV diagnosis period [n = 707]**				
1986–2004	199 (28)	193 (42)	6 (2)	**<0.001**
2005–2009	273 (39)	202 (44)	71 (29)	
2010–2013	235 (33)	63 (14)	172 (69)	
**Newly diagnosed [n = 707]**				
≤ 1 year	218 (31)	20 (4)	198 (80)	**<0.001**
> 1 year	489 (69)	438 (96)	51 (20)	
**Mode of transmission [n = 402]**				
MSM	85 (21)	24 (11)	61 (34)	**<0.001**
Heterosexual	317 (79)	196 (89)	121 (66)	
**Clinical condition [n = 598]**				
Asymptomatic / acute	381 (64)	184 (48)	197 (92)	**<0.001**
AIDS	217 (36)	200 (52)	17 (8)	
**Viral load (RNA copies/ml) [n = 674]**				
< 10,000	162 (24)	114 (26)	48 (21)	0.200
10,000–100,000	321 (48)	210 (48)	111 (48)	
> 100,000	191 (28)	117 (26)	74 (32)	
**CD4+ T count (cells/μl) [n = 605]**				
≥ 500	98 (16)	23 (6)	75 (33)	**<0.001**
200–499	225 (37)	131 (34)	94 (42)	
< 200	282 (47)	227 (60)	55 (25)	

Data are number (%). Significant differences (***p***<0.05) are marked in bold.

### Prevalence and patterns of ADR-CRM and SDRM

Tables 2 and 3 summarize the epidemiological and clinical information of subjects with and without ADR-CRM or SDRM, respectively. Of the 717 studied subjects, 432 (60%) had ADR-CRM or SDRM: 87.5% of ARV drug-experienced subjects and 9.2% of ARV drug-naïve subjects. Individuals at the AIDS stage (*p* = 0.007), with lower CD4+ T cell count (*p* = 0.002) and higher viral load (*p* = 0.021) were more likely to present ADR-CRM (Table 2 and S1 Table). The length of time under ART, by contrast, was not associated with the presence of ADR-CRM (*p* = 0.087) (Table 2 and S1 Table). No significant differences were observed between subjects with and without SDRM for the analyzed demographic and clinical variables (Table 3).

**Table 2:** Clinical data of ARV drug-experienced Panamanian HIV-1 subtype B subjects according to presence or absence of CRM resistance mutations to ARV inhibitors.

Feature	Total	ADR-CRM	non ADR-CRM	
	N = 467 (%)	n = 409 (%)	n = 58 (%)	*p*-value
**Gender**				
Male	311 (67)	294 (67.1)	46 (61.3)	0.396
Female	156 (33)	144 (32.9)	29 (38.7)	
**Age group (years)**				
< 25	27 (6)	12 (3)	15 (26)	**<0.001**
25–44	301 (64)	271 (66)	30 (52)	
45–76	139 (30)	126 (31)	13 (22)	
**HIV diagnosis period [n = 458]**				
1986–2004	193 (42)	174 (43)	19 (34)	0.075
2005–2009	202 (44)	178 (44)	24 (43)	
2010–2013	63 (14)	50 (12)	13 (23)	
**Genotyping performed by time of new ART inclusion**				
2008–2010	96 (21)	80 (20)	16 (28)	0.156
2011–2013	371 (79)	329 (80)	42 (72)	
**Number of newly diagnosed [n = 458]**				
≤ 1 year	20 (4)	9 (2)	11 (20)	**<0.001**
> 1 year	438 (96)	393 (98)	45 (80)	
**Clinical condition [n = 384]**				
Asymptomatic / acute	184 (48)	154 (45)	30 (67)	**0.007**
AIDS	200 (52)	185 (55)	15 (33)	
**Subjects presently under ART [n = 460]**				
Yes	439 (95)	397 (98)	42 (76)	**<0.001**
No	21 (5)	8 (2)	13 (24)	
**Time under ART (years) [n = 406]**				
≤3 years	144 (35)	121 (34)	23 (50)	0.087
3.1–5.9 years	111 (27)	102 (28)	9 (20)	
≥6 years	151 (37)	151 (38)	14 (30)	
**CD4+ T cell count (cells/μl) [n = 381]**				
≥ 500	23 (6)	15 (4)	8 (17)	**0.002**
200–499	131 (34)	114 (34)	17 (36)	
< 200	227 (60)	205 (61)	22 (47)	
**Viral load (RNA copies/ml plasma) [n = 441]**				
< 10,000	114 (26)	91 (24)	23 (41)	**0.021**
10,000–100,000	210 (48)	189 (49)	21 (38)	
> 100,000	117 (26)	105 (27)	12 (21)	
**Subjects with mutations according to ARV class of inhibitor**				
NRTI	14 (3)	11 (3)	3 (5)	**<0.001**
NNRTI	37 (8)	33 (8)	4 (7)	
PI	17 (4)	2 (0.5)	15 (26)	
Mutations to ≥2 inhibitors	363 (78)	363 (89)	0 (0)	
No DR-associated polymorphisms	36 (8)	0 (0)	36 (62)	
**Subtype B variants**				
B_PANDEMIC_	430 (92)	376 (92)	54 (93)	0.961
B_CARIBBEAN_	37 (8)	33 (8)	4 (7)	

Data are number (%). ADR-CRM, acquired ARV drug resistance mutation of clinical relevance. ART, antiretroviral therapy. NRTI, nucleoside reverse transcriptase inhibitors. NNRTI, non-nucleoside reverse transcriptase inhibitors. PI, protease inhibitors. Significant differences (***p***<0.05) are marked in bold.

**Table 3:** Clinical data of ARV drug-naïve Panamanian HIV-1 subtype B subjects with and without SDRM.

Feature	ART drug-naïve	SDRM	no SDRM	
	N = 250 (%)	n = 23 (%)	n = 227 (%)	*p-*value
**Gender**				
Male	169 (68)	17 (74)	152 (67)	0.700
Female	81 (32)	6 (26)	75 (33)	
**Age group (years)**				
< 25	103 (41)	10 (43)	93 (41)	0.815
25–44	120 (48)	10 (43)	110 (48)	
45–76	27 (11)	3 (13)	24 (11)	
**HIV diagnosis by time of new ART introduction [n = 249]**				
1998–2006	12 (5)	0 (0)	12 (5)	0.295
2007–2010	107 (43)	8 (35)	99 (44)	
2011–2013	130 (52)	15 (65)	115 (51)	
**Newly diagnosed [n = 249]**				
≤ 1 year	198 (80)	21 (91)	177 (78)	0.141
> 1 year	51 (20)	2 (9)	49 (22)	
**Mode of transmission [n = 182]**				
MSM	61 (34)	5 (33)	56 (34)	0.987
Heterosexual	121 (66)	10 (67)	111 (66)	
**Clinical condition [n = 214]**				
Asymptomatic / acute	197 (92)	19 (95)	178 (92)	0.609
AIDS	17 (8)	1 (5)	16 (8)	
**CD4+ T cell count (cells/μl) [n = 224]**				
≥ 500	75 (33)	5 (25)	70 (34)	0.480
200–499	94 (42)	8 (40)	86 (42)	
< 200	55 (25)	7 (35)	48 (24)	
**Viral load (RNA copies/ml plasma) [n = 233]**				
< 10,000	48 (21)	4 (17)	44 (21)	0.720
10,000–100,000	111 (48)	10 (43)	101 (48)	
> 100,000	74 (32)	9 (39)	65 (31)	
**Subjects with mutations according to ARV class of inhibitor [n = 250]**				**<0.001**
NRTI	7 (3)	7 (30)	0 (0)	
NNRTI	41 (16)	9 (39)	32 (14)	
PI	62 (25)	3 (13)	59 (26)	
Mutations to ≥2 inhibitors	4 (2)	4 (17)	0 (0)	
No DR-associated polymorphisms	136 (54)	0 (0)	136 (60)	
**Subtype B variants**				
B_CARIBBEAN_	8 (3)	1 (4)	7 (3)	0.774
B_PANDEMIC_	242 (97)	22 (96)	220 (97)	

Data are number (%). SDRM, surveillance drug resistance mutations. ART, antiretroviral therapy. NRTI, nucleoside reverse transcriptase inhibitors. NNRTI, non-nucleoside reverse transcriptase inhibitors. PI, protease inhibitors. Significant differences (***p***<0.05) are marked in bold.

**Fig 1:**
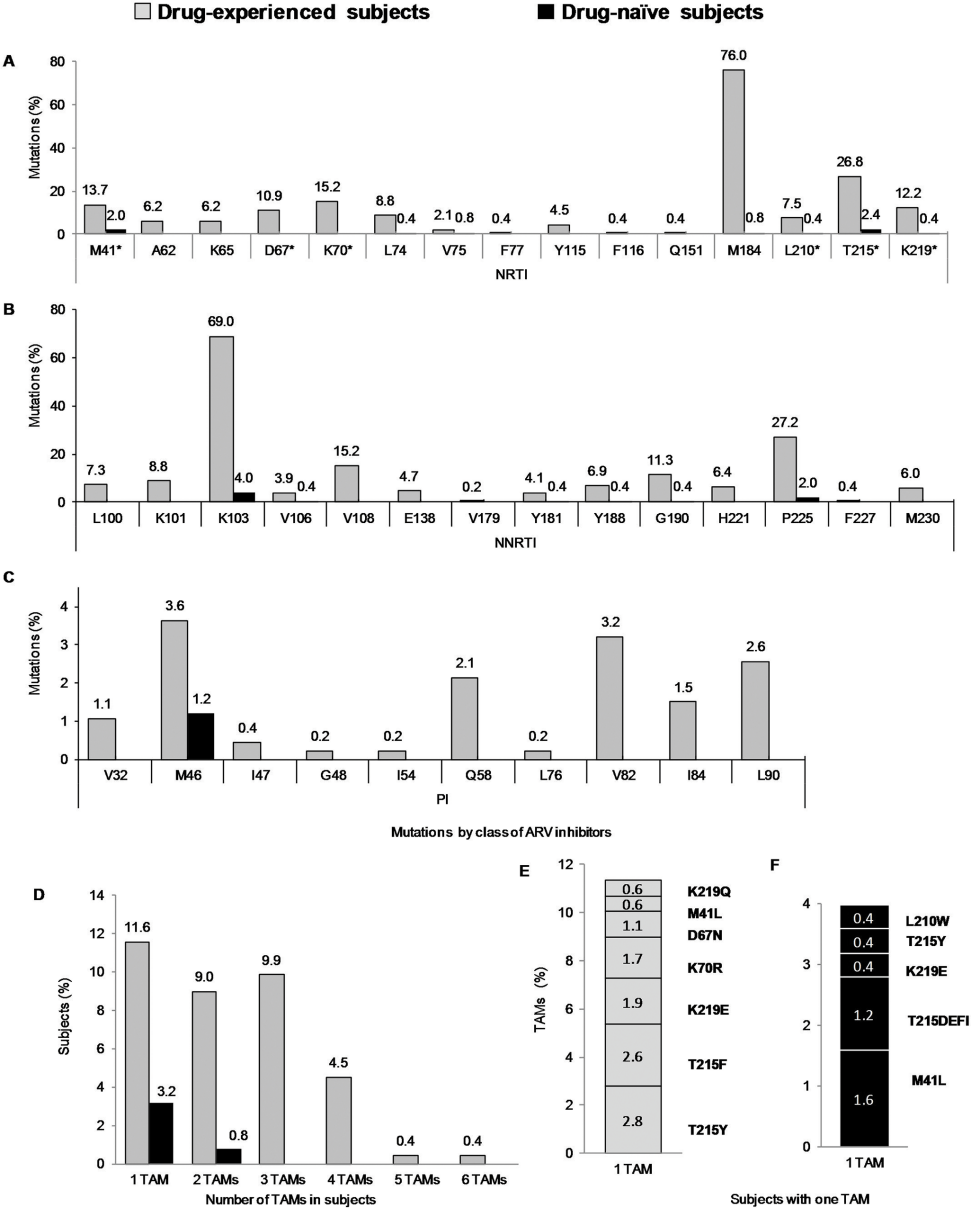
ADR-CRM and SDRM comparison by class of ARV inhibitors and of Tymidine-Analog-Mutations (TAM's) associated to nucleoside reverse transcriptase inhibitors (NRTI) in ARV drug-experienced subjects (*n* = 467) and ARV drug-naïve subjects (n = 250). **A**, ADR-CRM and SDRM frequency associated to nucleoside reverse transcriptase inhibitor (NRTI); TAMs position are indicated by an asterisk. **B**, ADR-CRM and SDRM frequency associated to non-nucleoside reverse transcriptase inhibitor (NNRTI). **C**, ADR-CRM and SDRM frequency associated to protease inhibitor (PI). **D**, Proportion of subjects harboring one or more TAM according to subject´s drug use status. **E**, Frequency of mutations in ARV drug-experienced subjects with only one TAM. **F**, Frequency of mutations in ARV drug-naïve subjects with only one TAM. Graph bars colors are according to legend at top.

The majority of subjects with ADR-CRM (78%) had a pattern of mutations that confer resistance to more than two classes of ARV inhibitors (Table 2 and S1 Table), whereas most subjects with SDRM, had mutations mainly to a single class drugs (NRTI or NNRTI) (Table 3). The patterns and frequency of ADR-CRM and SDRM associated with each ARV drug class are showed in Fig 1A–1C. For NRTIs, the most common ADR-CRM were M184VI (76.0%) and T215YF (26.8%), whereas the most common SDRM were T215 revertants (2.4%) and M41L (2.0%) (Fig 1A). For NNRTIs, the most common ADR-CRM and SDRM were K103N (69.0% and 4.0%, respectively) and P225H (27.2% and 2.0%, respectively) (Fig 1B). For PIs, the most frequent ADR-CRM were M46IL (3.6%), V82AT (3.2%), L90M (2.6%) and Q58E (2.1%) and, the only SDRM observed was M46L/I (1.5%) (Fig 1C).

Of the 467 ARV drug-experienced and of the 250 ARV drug-naïve subjects, 167 and 10 subjects harbored ***tymidine-analog-mutations*** (TAMs), respectively. In ARV drug-experienced subjects, TAMs found were T215Y/F (26.8%), K70R (15.2%), M41L (13.7%), K219E/Q (12.2%), D67N (10.9%), and L210W (7.5%) (Fig 1A). In ARV drug-naïve subjects, observed TAMs were T215rev (2.0%), T215F (0.4%), M41L (2.0%), K219EQ (0.4%) and L210W (0.4%) (Fig 1A). Among subjects with TAMs, a high percentage of ARV drug-naïve (80%, 8/10) and ARV drug-experienced subjects (34%, 54/167) harbored one TAM (Fig 1D). In ARV drug-experienced subjects presenting only one TAM, mutations T215Y (2.8%) and T215F (2.6%) were the most common; whereas, M41L (1.6%) was predominant in ARV drug-naïve subjects (Fig 1E and 1F). Only two ARV drug naïve subjects harbored two TAMs (M41L and T215FD), whereas a substantial proportion of ARV drug-experienced subjects harbored two or three TAMs (Fig 1D). Of the 42 ARV drug-experienced subjects with two TAMs, 11 different TAM combinations were observed; being TAMs combinations M41L + T215Y (40%) as the most common, followed by M41L + T215F (14%). Fifteen combinations were observed among 46 ARV drug-experienced subjects with 3 TAMs; where 39% of these subjects harbored the combination M41L + T215Y + L210W, followed in 11% by combination of D67N + K70R + K219Q.

### ART schemes and associated patterns of ADR-CRM

**Fig 2:**
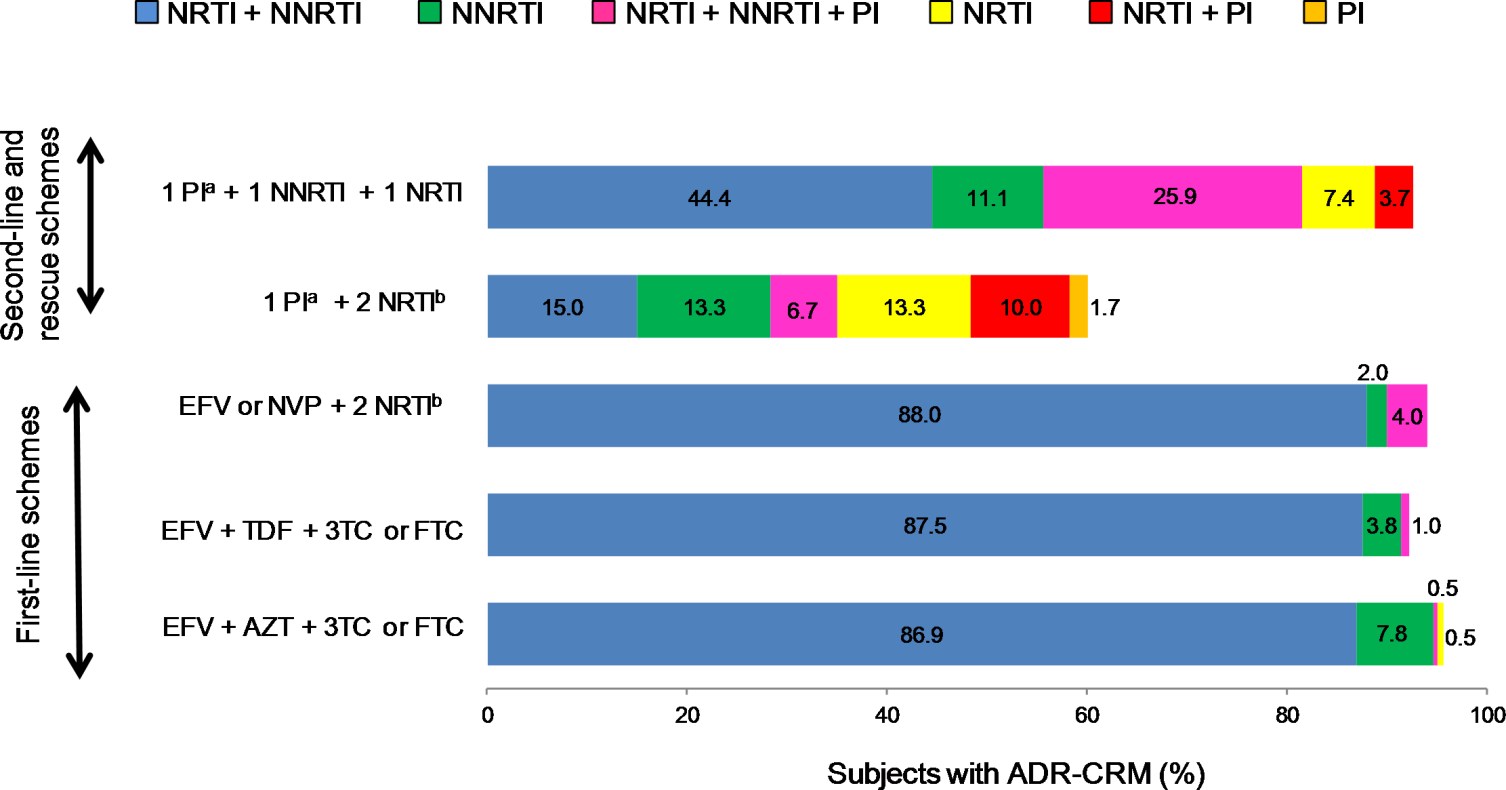
ADR-CRM present in ARV drug-experienced subjects (n = 467) in use of or exposed to the specified ART scheme recommended as first-line, second-line or rescue-line schemes. Proportion of subjects with ADR-CRM to nucleoside reverse transcriptase inhibitor (NRTI), non-nucleoside reverse transcriptase inhibitor (NNRTI) or protease inhibitors (PI) (as explained at top right legend) under or exposed to ART schemes EFV+AZT+3TC or FTC (*n* = 206), EFV+TDF+3TC or FTC (*n* = 104), EFV or NVP plus any NRTI (*n* = 50), PI plus two NRTI (*n* = 60) and PI-based rescue-schemes (*n* = 27). ^**a**^, PI: ATV, IDV/r, NFV/r, DRV/r, LVP or LPV/r. ^**b**^, NRTI: any combination of DDI, D4T, 3TC, FTC, AZT.

**Fig 3:**
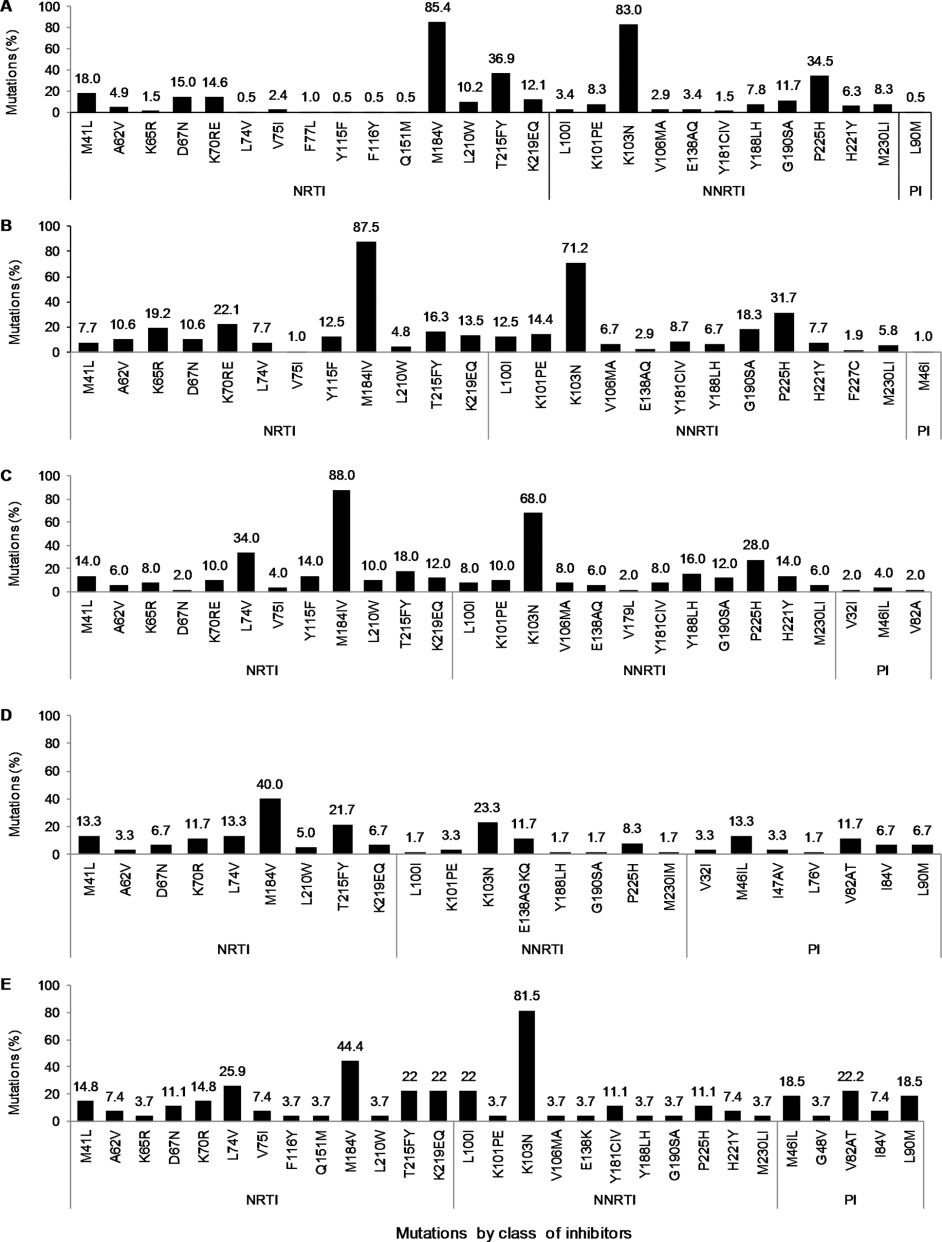
Frequency and patterns of ADR-CRM to ARV drugs class of reverse transcriptase (NRTI/NNRTI) and protease inhibitors (PI) in ARV drug-experienced subjects (n = 467) according to ART scheme. **A**, Subjects under or exposed to first-line scheme EFV + AZT + 3TC or FTC (n = 206). **B**, Subjects under or exposed to first-line scheme EFV + TDF + 3TC or FTC (n = 104). **C**, Subjects under or exposed to alternative first-line scheme EFV or NVP + 2 NRTI^b^ (n = 50). **D**, Subjects under or exposed to second-line scheme 1 PI^a^ + 2 NRTI^b^ (n = 60). **E**, Subjects under or exposed to rescue-line scheme 1 PI^a^ + 1 NNRTI + 1 NRTI (n = 27). ^**a**^, PI: ATV, IDV/r, NFV/r, DRV/r, LVP or LPV/r. ^**b**^, NRTI: any combination of DDI, D4T, 3TC, FTC, AZT.

The present cohort of 467 ARV drug-experienced subjects included individuals under first line-ART schemes EFV+AZT+3TC or FTC (*n* = 206), EFV+TDF+3TC or FTC (*n* = 104), EFV or NVP plus any NRTI (*n* = 50); second line-schemes consisting of one PI plus two NRTIs (*n* = 60) and PI-based rescue-schemes (*n* = 27). Twenty subjects were under ART, but information on ART schemes was unavailable. Incomplete viral suppression (95%, 443/467) was the reason why most of the subjects were referred for HIV genotypic drug resistance testing and most subjects showed ADR-CRM for the ART scheme under current use (Fig 2). Subjects using first line-schemes harbored mainly ADR-CRM (>85%) to NRTIs combined with NNRTIs, whereas mixed patterns of ARV inhibitors combinations were observed among subjects under second-line and rescue schemes (Fig 2). Fig 3 describes the frequency and patterns of drug resistance mutations according to ARV drug class and ART scheme in use. The frequency and patterns of mutations differed slightly among individuals under first-line ART schemes; however, in all cases higher percentages of M184VI, K103N and P225H were observed (Fig 3A, 3B and 3C). Second-line scheme showed lesser number and lower frequency of mutations for NRTIs and NNRTIs compared to first-line and rescue-line schemes (Fig 3A–3E). The highest percentage of NRTIs mutations in subjects on second-line scheme were M184V and T215FY in only 40.0% and 21.7% of the subjects compared to up to 88.0% and 83.0% respectively observed in subjects on other schemes. In addition, K103N frequency was 23.3% in subjects under second-line scheme, the lowest observed among all schemes (Fig 3A–3E). Mutations associated with PIs were more observed at position M46IL and V82A in subjects on second-line and rescue schemes (Fig 3D and 3E).

### ARV treatment regimens and prevalence of SDRM and ADR-CRM

**Fig 4:**
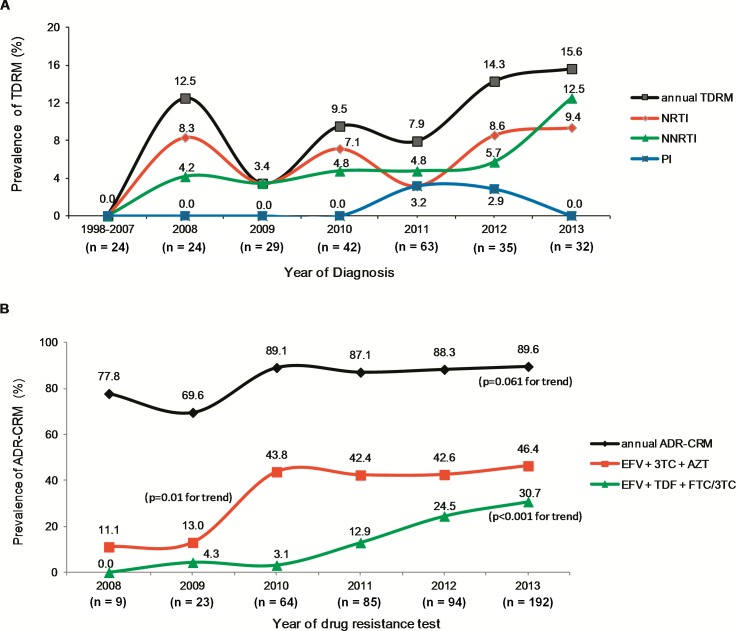
Annual prevalence of SDRM and acquired drug resistance ADR-CRM in ARV drug-naïve and ARV drug-experienced Panamanian HIV-1 subtype B infected subjects. **A**, Prevalence of SDRM by year of diagnosis from 1998 to 2013 in ARV drug-naïve subjects (n = 250). **B,** Prevalence of ADR-CRM by date of sampling from 2008 to 2013 in ARV drug-experienced subjects (n = 467).Graph line colors are according to legend at right respectively. Only statistically significant *p* values are shown.

During the studied period, no statistically significant difference were detected in annual SDRM (*p* = 0.556) or ADR-CRM (*p* = 0.156) prevalence despite of changes in ART schemes in 2007 and 2011 (Tables 2 and 3). Annual SDRM prevalence tended to rise from 0.0% in 1998–2007 up to 15.6% in 2013, but without a statistically significant trend (*p* = 0.095) (Fig 4A). The percentage of subjects with NRTI associated SDRM (SDRM-NRTI) fluctuated between years and showed a decrease of 3.2% in 2011 followed by a rise to 9.4% in 2013. The percentage of subjects with NNRTI associated SDRM (SDRM-NNRTI) was similar during initial years, but increased steeply from 5.7% in 2012 to 12.5% in 2013; however, without a statistically significant trend (*p* = 0.069). After the latest introduction of ART in 2011, SDRM-NRTIs and SDRM-NNRTIs increased in the following years (Fig 4A). Annual ADR-CRM prevalence showed an increment of 11.8% in six years without a statistically significant trend (*p* = 0.061) (Fig 4B). Prevalence of ADR-CRM showed a statistically significant increase in subjects under the use of EFV + 3TC + AZT (p = 0.010) and EFV + TDF + FTC/3TC (*p* < 0.001) (Fig 4B).

### HIV-1 subtype B clades and prevalence of SDRM and ADR-CRM

To understand the role of viral diversity in the Panamanian HIV-1 epidemic, subjects with and without SDRM or ADR-CRM were analyzed according to the subtype B lineages classification. ML phylogenetic analysis of Panamanian HIV-1 subtype B *pol* sequences determined that 672 (94%) subjects branched within the B_PANDEMIC_ clade; whereas the remaining 45 (6%) were among the B_CARIBBEAN_ lineages. We observed not significant difference in the prevalence of SDRM (p = 0.774) and ADR-CRM (p = 0.961) across different subtype B viral clades (Tables 2 and 3).

### Predicted ARV susceptibility in ARV drug-experienced and ARV drug-naïve subjects

**Fig 5:**
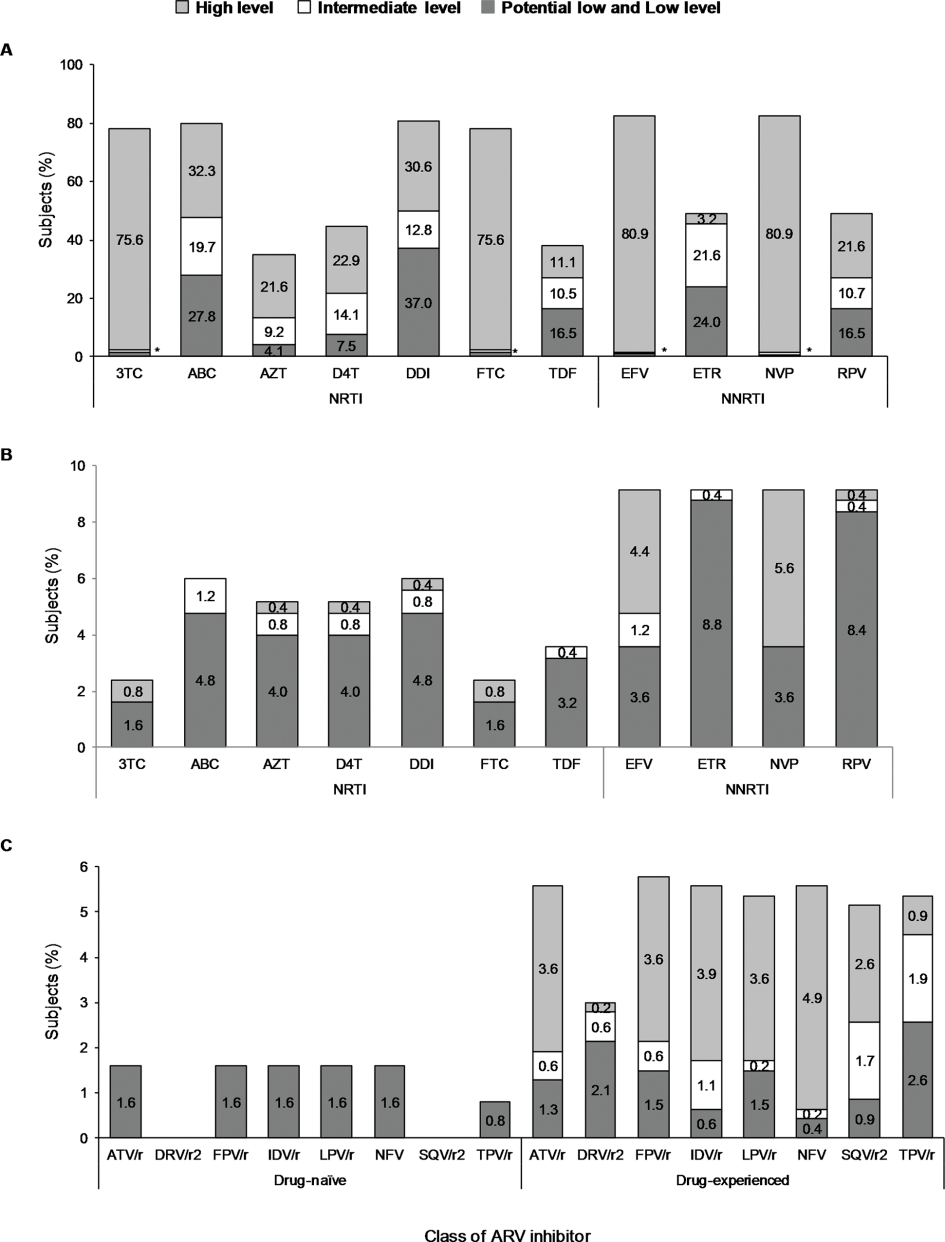
Level of ARV drug resistance according to each class of reverse transcriptase and protease inhibitors in Panamanian HIV-1 subtype B infected subjects. **A**, Level of ARV drug resistance to nucleoside reverse transcriptase inhibitor (NRTI) and non-nucleoside reverse transcriptase inhibitor (NNRTI) in ARV drug-experienced subjects (n = 467). **B**, Level of ARV drug resistance to nucleoside reverse transcriptase inhibitor (NRTI) and non-nucleoside reverse transcriptase inhibitor (NNRTI) in ARV drug-naïve subjects (n = 250). **C**, Level of ARV drug resistance to protease inhibitor (PI) in ARV drug-naïve and ARV drug-experienced subjects. *, means frequency of subjects with <3%. Bar colors are according to legend at top.

High-level resistance to 3TC, FTC, EFV and NVP was observed in 75.6%, 75.6%, 80.9% and 80.9% of ARV drug-experienced subjects, respectively (Fig 5A). Conversely, ARV drug-naïve subjects showed potential-low and low-level resistance to NRTIs in less than 6%, and high-level resistance to EFV and NVP in 4.4% and 5.6%, respectively (Fig 5B). High-level of resistance to most PIs was observed in less than 2% of the ARV drug-naïve subjects, whereas, in ARV drug-experienced subjects was between 0.9% for TPR/r to 4.9% for NFV (Fig 5C).

## Discussion

In this study, we assessed the prevalence and patterns of ADR-CRM and SDRM that conferred resistance to RT and/or PI inhibitors, over a 5-year period, in HIV-1 subtype B-infected subjects from Panama. We report here an overall prevalence of ADR-CRM of 87.6% and of TDR of 9.2%. This represents a sharp increase respect to the first study conducted in Panama between 2004 and 2005 that revealed an overall prevalence of ADR-CRM of 9.7% and no SDRM [12]. The lowest frequency of NNRTIs and NRTIs associated mutations for 2004 to 2005 period in both ARV drug-experienced and ARV-naïve subjects were probably due to the short-term uses and low coverage of ARV of that time [12, 26, 27].

Although identifying the recently-infected condition of the ARV drug-naïve subjects was a limitation in our study, our resulted SDRM prevalence (9.2%) was slightly lower than that previously estimated (12.8%) for Panama [13], using the WHO criteria for recently-infected subjects [13, 28]. Thus there is possibility that the resistance associated mutations in ARV drug-naïve subjects evaluated in our study may not be overestimated due to ARV-naïve misclassification. The SDRM prevalence observed in Panama is comparable to those recently described in other Latin American countries that also reported intermediate levels of TDRM in most cases: 5.7% in El Salvador [29], 5.8% in Colombia [30], 6.8% in Mexico [31], 7.0% in Honduras [32], 7.3%-8.3% in Guatemala [33, 34], and 8.1% in Brazil [35]. The continuous molecular epidemiological surveillance of HIV-1 Latin American epidemics is crucial to determine whether TDRM prevalence in this region will remain stable or not in the following years.

In Panama, changes in SDRM patterns and level of resistance had occurred over time. First Panamanian study showed no TDR mutations in 2005 [12], but in our study SDRM-NNRTIs prevalence (7.6%) was higher than SDRM-NRTIs (6.4%) and SDRM-PIs (1.2%) prevalence. Our mutation pattern analyses showed that the most frequent SDRM were: K103N (4.0%) and P225H (2.0%) for NNRTIs, T215 revertants (2.4%) and M41L (2.0%) for NRTIs, and M46L/I (1.2%) for PIs. Thus, ARV drug-naïve population in our study showed higher levels of resistance to EFV and NVP than to others ARVs.

We also detected a high level of resistance to EFV and NVP in 80.9% of the ARV drug-experienced subjects. There were 77% of the ARV drug-experienced subjects under EFV-based regimens, which have been used preferentially as first line-schemes in Panama since 2007 [8, 10]. The pattern of mutations associated with NNRTIs in ARV drug-experienced subjects on first line-schemes reveals a high prevalence of mutations K103N (69.0%) and P225H (27.2%). Previously reported NNRTIs mutations for the 2004 to 2005 period in ARV drug-experienced subjects exposed to AZT or d4T + 3TC or ddI + EFV or NFV or IDV reported K103N at a lower frequency (4.9%), together with mutations L100I (1.2%) and Y318F (1.2%) [12]. Mutations K103N and P225H are preferentially selected by EFV, which is a second generation NNRTI [36].

In the studied ARV drug-experienced subjects, other major resistance mutations that are selected by first generation NNRTIs (NVP and DLV) and confer high level of resistance with clinical failure were found in lower frequency (K101, V106, Y181, Y188, G190, F227 and E138) [37–39]. Nevertheless, our study revealed a low prevalence of mutations at positions L100, K101, V179, Y181, G190, F227 and M230 which may contribute to reduced susceptibility to all NNRTIs, including third generation drugs ETR and RVP [40]. These two ARV drugs are administered alternatively when K103N, V106A/M and P225H are already expressed, as these mutations are not selected by these drugs [40].

Intermediate to high resistance levels to NRTIs in the ARV drug-experienced subjects were reported in Panama in previous years (2004–2005) and were associated to mutations L74V (2.4%), T215F/Y (3.6%) and M184V (1.2%) [12]. In our study, the most frequent NRTIs associated mutation was M184V (76%), followed by T215F/Y (26.8%). L74V was the second most frequent mutation in subjects using the alternative first line-scheme consisting of EFV + 2 NRTIs, mainly ABC (37.3%) or ddI (23.5%) or AZT (19.6%). M184V is selected during 3TC and FTC treatment and has been shown to confer high-level resistance to these drugs [41]. Therefore, the observed high level of resistance to 3TC and FTC in 75.6% of ARV drug-experienced subjects may be explained by the high frequency of M184V, irrespective of ART scheme in use. In addition, T215F/Y confers high-level resistance to AZT and d4T and varying degrees of resistance to most NRTIs [42, 43].

TAM prevalence ranged from 4.7% to 17.5% in drug-experienced and from 0.4% to 2.2% in drug-naïve subjects. The high number of subjects with one or more TAMs in a population decreases the predicted success of novel NRTIs currently in clinical trials such as 4´-ethynyl-2-fluoro-2´-deoxyadenosine (EFdA) and d4T derivative 2´,3´-didehydro-3´-deoxy-4´-ethynylthymidine (Ed4T) [44, 45]. Our results also show a high percentage of ARV drug-experienced subjects with two (9.0%) or three (9.9%) TAMs. Thus, the impact of TAMs need to be better evaluated in coming years since HIV-infected subjects with mutations M184V alone, P119S/T165A/M184V combined or T69 insertion complex, including T210W and T215Y, may have significantly decreased susceptibility to the novel drug Ed4T [45].

Although it seems that combination ARV regimens are effective against all HIV-1 subtypes and recombinants, there is emerging evidence of subtype differences in drug resistance mutations prevalence and patterns [2, 46, 47]. Even though the Panamanian epidemic is driven predominantly by subtype B, both B_PANDEMIC_ and B_CARIBBEAN_ clades has been detected circulating in this country [20]. Our findings show no difference in ADR-CRM and SDRM prevalence or patterns between subjects infected with different subtype B clades. To better understand if ART regimes may be influenced by the HIV-1 subtype B genetic diversity, a study should be conducted in the Caribbean region where the highest prevalence of HIV-1 B_CARIBBEAN_ clades has been reported [48].

In our study, neither overall ADR-CRM nor SDRM prevalence increased according to each studied period and both remained indistinctly despite of changes in guidelines for prescription of ART schemes occurred in 2007 and 2011. However, ADR-CRM increased in subjects under the first-line scheme AZT + 3TC + EFV in 2009 and under FTC + TDF + EFV since 2011. Interestedly, SDRM prevalence for the studied period of 1998 to 2013 appears to show a possible upward trend in the case of the SDRM-NNRTIs but a fluctuating pattern for TDR-NRTIs. Then, the increase of ADR-CRM prevalence associated to first-line schemes may be partly explained by the long-term use of first and second generation NNRTIs in Panama. Low ART-adherence, ARV toxicity, discontinued or intermittent access to ARV drugs, suboptimal dosage and long duration of virological failure in individuals on first line-schemes have been factors associated to ARV drug resistance over time [49, 50].

In summary, the increment of ADR-CRM for the recommended first-line schemes and the likely imminent increase in SDRM-NNRTI observed over the past four years suggest a reduced efficacy of EFV based regimens in the coming years. Consequently, greater transmission of HIV-1 could be expected as the effective infection depends strongly on individual levels of viremia and thereby drug-resistant viruses are mainly transmitted from treated subjects with virological failure [51–53]. Although we conducted a retrospective study using convenience sampling method, these results provide important information to make effective public health decisions on selecting the best ART regimens for Panamanian population living with HIV.

## Supporting Information

S1 Table Univariate and multiple logistic regression analysis for CRM mutation. (DOCX)
