# Supplementary material for: HIV-1 Antiretroviral Drug Resistance Mutations in Treatment Naïve and Experienced Panamanian Subjects: Impact on National Use of EFV-Based Schemes
Source: PLoS One. 2016 Apr 27;11(4):e0154317. doi: 10.1371/journal.pone.0154317 (PMC4847863; doi:10.1371/journal.pone.0154317)
Supplement: S1 Table — (DOCX) [file pone.0154317.s001.docx]

S1 Table. Univariate and multiple logistic regression analysis for CRM mutation.

|  | Univariate Logistic Analysis | | |  | Multiple Logistic Analysis | | |
| --- | --- | --- | --- | --- | --- | --- | --- |
| **Feature** | **OR** | **(95% CI)** | **p-value** |  | **AOR** | **(95% CI)** | **p-value** |
| **Gender** |  |  |  |  |  |  |  |
| Male | Ref. |  |  |  | Ref. |  |  |
| Female | 0.95 | (0.53-1.69) | 0.928 |  | 0.92 | (0.51-1.68) | 0.797 |
| **Age group (years)** |  |  |  |  |  |  |  |
| < 25 | Ref. |  |  |  | Ref. |  |  |
| 25-44 | **11.29** | **(4.84-26.35)** | **<0.001** |  | **10.80** | **(4.45-26.25)** | **<0.001** |
| 45-76 | **12.11** | **(4.69-31.32)** | **<0.001** |  | **11.69** | **(4.24-32.20)** | **<0.001** |
| **Clinical condition** |  |  |  |  |  |  |  |
| Asymptomatic / acute | Ref. |  |  |  | Ref. |  |  |
| AIDS | **2.40** | **(1.25-4.63)** | **0.009** |  | **2.42** | **(1.24-4.72)** | **0.010** |
| **Subjects presently under ART** |  |  |  |  |  |  |  |
| No | Ref. |  |  |  | Ref. |  |  |
| Yes | **15.36** | **(6.02-39.18)** | **<0.001** |  | **15.03** | **(5.69-39.72)** | **<0.001** |
| **Time under ART (years)** |  |  |  |  |  |  |  |
| ≤3 years | Ref. |  |  |  | Ref. |  |  |
| 3.1 - 5.9 years | 2.15 | (0.95-4.86) | 0.065 |  | 2.24 | (0.93-5.36) | 0.071 |
| ≥6 years | 1.86 | (0.92-3.78) | 0.086 |  | 1.91 | (0.76-4.77) | 0.167 |
| **CD4+ T cell count (cells/μl)** |  |  |  |  |  |  |  |
| ≥ 500 | Ref. |  |  |  | **Ref.** |  |  |
| 200-499 | 0.87 | (0.38-1.98) | 0.734 |  | 0.86 | (0.37-1.96) | 0.713 |
| < 200 | **0.22** | **(0.07-0.72)** | **0.012** |  | **0.20** | **(0.06-0.69)** | **0.011** |
| **Viral load (RNA copies/ml plasma)** | |  |  |  |  |  |  |
| < 10,000 | Ref. |  |  |  | Ref. |  |  |
| 10,000-100,000 | **2.27** | **(1.20-4.32)** | **0.012** |  | **2.46** | **(1.25-4.87)** | **0.010** |
| > 100,000 | **2.21** | **(1.04-4.69)** | **0.039** |  | **2.43** | **(1.11-5.34)** | **0.027** |
| **Subjects with mutations according to ARV class of inhibitor** | | |  |  |  |  |  |
| One mutation (NRTI or NNRT or PI) | Ref. |  |  |  | Ref. |  |  |
| Mutation to two or more inhibitors | **55.34** | **(15.72-194.87)** | **<0.001** |  | **51.11** | **(13.98-186.84)** | **<0.001** |
| **Subtype B variants** |  |  |  |  |  |  |  |
| B_PANDEMIC_ | Ref. |  |  |  | Ref. |  |  |
| B_CARIBBEAN_ | 1.18 | (0.40-3.48) | 0.757 |  | 1.30 | (0.43-3.92) | 0.637 |

OR, odds ratio; CI, confidence interval; AOR, adjusted odds ratio for gender, age in years, and HIV diagnosis period.
